# Long-term sequelae of sporadic cryptosporidiosis: a follow-up study

**DOI:** 10.1007/s10096-018-3268-9

**Published:** 2018-05-05

**Authors:** Zsófia Iglói, L. Mughini-Gras, L. Nic Lochlainn, A. Barrasa, J. Sane, S. Mooij, B. Schimmer, J. Roelfsema, W. van Pelt, T. Kortbeek

**Affiliations:** 1 National Institute for Public Health and the Environment (RIVM), Bilthoven, the Netherlands; 2 European Program for Public Health Microbiology Training (EUPHEM), European Centre for Disease Prevention and Control (ECDC), Stockholm, Sweden; 3 Faculty of Veterinary Medicine, Utrecht University, Utrecht, the Netherlands; 4 European Programme for Intervention Epidemiology Training (EPIET), European Centre for Disease Prevention and Control (ECDC), Stockholm, Sweden; 5 National Centre for Epidemiology, Madrid, Spain

**Keywords:** *Cryptosporidium hominis*, *Cryptosporidium parvum*, Case-crossover study, Long-term sequela, The Netherlands

## Abstract

To determine the frequency of occurrence of sequelae following cryptosporidiosis. A follow-up study was performed during a case-control study for sporadic cryptosporidiosis in the Netherlands (2013–2016). Cryptosporidiosis cases were invited to complete a follow-up questionnaire 4 months after diagnosis. Using a case-crossover study design, we compared the frequencies of reported symptoms 4 months after the acute phase to those reported 4 months before the onset of illness and during illness. Frequencies of symptoms in the pre- to post-infection phases were also compared with those of a population control group. *Cryptosporidium* species-specific effects were also studied. Logistic regression was used to calculate adjusted odds ratios (aOR) for symptoms occurrence. Of the 731 available cases, 443 (60%) responded and 308 (42%) could be included in the follow-up study. The median age was 26 years (range 1–80); 58% were female; 30% were infected with *C*. *hominis* and 70% with *C*. *parvum*. Compared to before illness, cases were significantly more likely to report dizziness (OR = 2.25), headache (OR = 2.15), fatigue (OR = 2.04), weight loss (OR = 1.82), diarrhoea (OR = 1.50), abdominal pain (OR = 1.38) or joint pain (OR = 1.84). However, symptoms of joint pain and headache occurred among cases after illness at a rate that was not significantly different from that observed in the general population. There were no significant differences in post-infection symptom occurrence between *C*. *hominis* and *C*. *parvum*. The disease burden of cryptosporidiosis extends beyond the acute phase of the infection, with cases reporting both intestinal and extra-intestinal symptoms up to 4 months following infection.

## Introduction

*Cryptosporidium* species are protozoan parasites that infect the epithelial cells of the gastrointestinal tract, causing gastrointestinal illness. Some species have zoonotic potential and their role was described in several large waterborne outbreaks worldwide [1–4]. In immunocompetent individuals, cryptosporidiosis is usually a self-limiting infection, with the most frequent symptoms being watery diarrhoea, nausea, vomiting, fever and abdominal pain, but infections may also be asymptomatic. However, severe diarrhoea and dissemination of the infection to extra-intestinal sites may occur in high-risk individuals, such as children, the elderly and immunocompromised individuals like HIV-infected persons [5]. There is no effective drug available to treat cryptosporidiosis.

Complications and long-term sequelae after an episode of acute gastroenteritis of infectious aetiology have been described for *Campylobacter jejuni*, *Escherichia coli*, most serotypes of *Salmonella enterica* [6], *Yersinia enterocolitica* [7], *Giardia lamblia* [8–10] and norovirus [11]. Besides considerable accumulated knowledge of the acute phase of cryptosporidiosis, there is growing evidence for the presence of long-term health implications following an acute episode of *Cryptosporidium* infection. In the Netherlands during 2007–2011, the burden of cryptosporidiosis expressed in disability-adjusted life years (DALY) was estimated at 75/year, but only (acute) diarrhoea was taken into account as a consequence of cryptosporidiosis [12]. However, reports of relapse or persisting diarrhoea, other gastrointestinal and non-gastrointestinal symptoms up to several months or even years post-infection are documented [13–16]. To our knowledge, only four epidemiological studies have investigated sequelae of *Cryptosporidium* infection. Rehn et al. [14] described the sequelae of cases involved in two cryptosporidiosis outbreaks caused by *C*. *hominis* up to 11 months after initial infection. Stiff et al. [13] investigated sequelae up to 1 year in a group of patients involved in an outbreak of *C*. *parvum* infection. While Hunter et al. [16] and Insulander et al. [15] investigated the role of both *C*. *hominis* and *C*. *parvum* (Insulander et al. looked at more than these two species) in sequelae amongst sporadic *Cryptosporidium* cases after 2 months of infection or following them up to 36 months respectively.

In the Netherlands, sporadic cryptosporidiosis is not notifiable by law and, in routine practice, *Cryptosporidium-*positive stools are not typed to determine the species. In the second half of 2012, an excess of cryptosporidiosis cases in the Netherlands, mainly due to *C*. *hominis* infection, triggered an international alert via the European Centre for Disease Control (ECDC)’s Epidemic Intelligence Information System (EPIS) for Food- and Water-borne Diseases (FWD). In response to this alert, the United Kingdom (UK) and Germany also reported an increase in cryptosporidiosis cases [17]. As no risk factors that could explain the observed increase were identified, a case-control study for sporadic cryptosporidiosis in the Dutch general population was conducted during 2013–2016 (article in preparation). This case-control study presented the opportunity to investigate possible long-term sequelae of sporadic cryptosporidiosis, including potential species-specific effects. A follow-up study was therefore conducted to determine the frequency of occurrence of several symptoms during and following an acute episode of *C*. *hominis* or *C*. *parvum* infection as compared with the period before such infection, as well as with the symptoms reported among population controls.

## Methods

### Data collection

During a case-control study to identify risk factors for sporadic cryptosporidiosis in the Netherlands (article in preparation), the participating cases were also invited via email to fill in a follow-up questionnaire 4 months after the onset of the acute phase of the infection. Recruitment of cases was performed through the collaboration with 17 regional Public Health Laboratories (PHLs) that perform *Cryptosporidium* diagnosis in the Netherlands. Cases were defined as those suffering from gastrointestinal complaints 2 weeks prior to sampling and having laboratory confirmation of *Cryptosporidium* spp. infection between April 2 2013 and April 1 2016. Controls were prospectively selected from the Netherlands’ population register by frequency matching for age and area of residence as done previously [18]. The expected number of cases (which the controls were frequency matched to) stratified by age was calculated using historic (2010–2012) data on cryptosporidiosis obtained from the participating 17 PHLs. Each control received an invitation letter explaining the study rationale and objectives, an informed consent, a control questionnaire and a prepaid envelope to return the questionnaire and informed consent to the Dutch National Institute for Public Health and the Environment (RIVM), where this study was performed. Questions in the questionnaires focused on musculoskeletal and other commonly described symptoms potentially associated with cryptosporidiosis, i.e. abdominal pain, vomiting, loss of appetite, weight loss, diarrhoea, headache, fatigue, dizziness, eye pain and joint pain. The follow-up questionnaire also contained a detailed list of body parts to narrow down the most affected joints, if any (i.e. neck, hips, shoulders, elbows, wrist, fingers, knees, ankles, heels, toes and upper and lower back). At follow-up, cases were asked to report whether the aforementioned symptoms were present 4 months after the acute phase of *Cryptosporidium* infection, but also whether they were already present 4 months before such infection. Within the framework of the case-control study, the same symptoms were also recorded during illness for the cases, as well as for the controls, which were then included in the analysis for comparison purposes. Basic demographic information was also collected. Returned questionnaires were encoded and their data coupled with the laboratory typing results.

### Data analysis

The study design was case-crossover [19, 20], in which we compared the frequencies of each reported symptom experienced during or 4 months after the acute phase of *Cryptosporidium* infection to those experienced 4 months before the acute illness. A complete-record analysis was performed using multivariable logistic regression models to calculate adjusted odds ratios (aOR) for the occurrence of each symptom (i.e. response variables) before vs. during or after *Cryptosporidium* infection (3-level categorical predictor variable), while adjusting for age (categorised as ≤ 5, 6–12, 13–25, 26–50 and ≥ 51 years), gender, *Cryptosporidium* species (*C*. *parvum* or *C*. *hominis*), season and study year. Clustering of observations (before, during and after illness) at the patient level was accounted for using cluster-robust standard errors. In addition, we compared the frequencies of each reported symptom in the cases before, during and after illness with those reported by the controls in the case-control study. To assess *Cryptosporidium* species-specific effects on the occurrence of each symptom, analyses were also carried out separately for *C*. *hominis* and *C*. *parvum*. Additional analyses were performed in which the age group of 0–5 years was excluded, as the quality of recording detailed symptoms in young children is likely to be lower. Data on the specific joints affected among those reporting joint pain as sequela were only available for the follow-up, i.e. 4 months after illness, but not for the periods during and before illness. While cases with travel history outside of the Netherlands were excluded from the case-control study, they were retained in this follow-up study, as whether a patient was infected domestically or abroad was irrelevant for the purposes of the present study. Controls were excluded if they had diarrheal illness 2–4 weeks before completing the questionnaire. All analyses were performed using STATA 14 (StataCorp LLC, College Station, USA).

### Genotyping

*Cryptosporidium* species was determined in all samples by using a real-time duplex PCR with dual labelled probes on a Roche LightCycler 480 apparatus using a combination of a PCR on *C*. *parvum* and a PCR developed specifically for *C*. *hominis*. The *C*. *parvum*-specific PCR targets a gene for a hypothetical protein and the *C*. *hominis* PCR targets part of the GP60 gene [21].

## Results

**Fig. 1:**
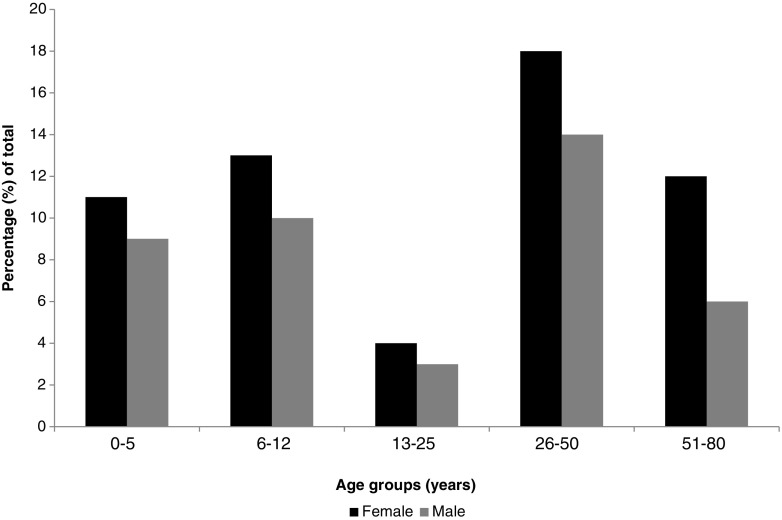
Distribution of the 308 cryptosporidiosis cases by age group and gender

Of the 731 cases included in the case-control study, 443 (60%) participated in the follow-up. Participation varied over the three study years (42, 25 and 33%, respectively). After exclusion of cases with missing data, a total of 308 (42%) cases were included in the follow-up study. The median age of these cases was 26 years (range 1 to 80 years), with the age group of 13–25 years being the least represented (Fig. 1). There were more female cases (58%), regardless of the infecting species in question: *C*. *hominis* (59%) and *C*. *parvum* (58%). Overall, 70% of cases were infected with *C*. *parvum* and the rest (30%) with *C*. *hominis*; the species distribution in the follow-up study over the three study years was 15, 13 and 61% for *C*. *hominis*, and 85, 87 and 39% for *C*. *parvum*.

**Fig. 2:**
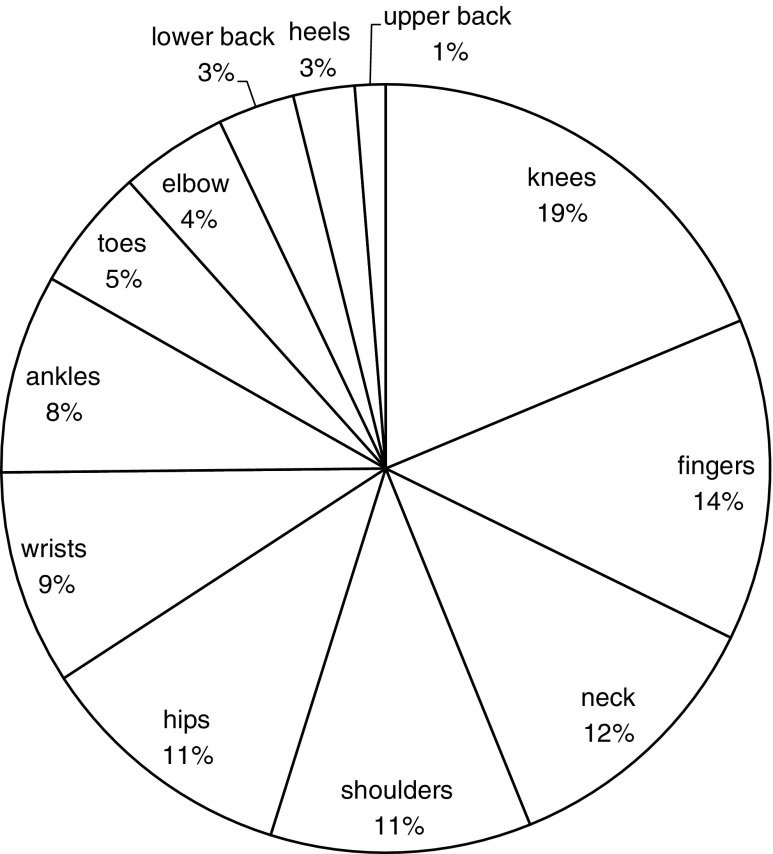
Proportion (%) of cases with pain in specific joints following cryptosporidiosis (*n* = 52)

Table 1 shows the frequencies of symptom occurrence among cryptosporidiosis cases during and after illness in comparison with the period before illness. The three most commonly reported symptoms 4 months after diagnosis were, in decreasing order fatigue (50.7%), diarrhoea (41.9%) and abdominal pain (38.3%). During illness, all symptoms were more prevalent and significantly more likely to occur than in the period before illness, with symptoms like loss of appetite, vomiting, diarrhoea, weight loss and abdominal pain being the obvious result of the (severe) episode of acute gastroenteritis. Apart from loss of appetite, eye pain and vomiting, all other symptoms were also significantly more likely to occur after acute illness as compared to the period before illness (Table 1). When patients were asked which symptoms got worse after the course of illness, fatigue (9%), joint pain (4%) and abdominal pain (3%) were those most commonly mentioned. Regarding joint pain, the most affected joints were the knees (19%), fingers (14%) or neck (12%) (Fig. 2), and often more than one joint was affected (67% of patients had complaints in multiple joints).

**Table 1:** Occurrence of symptoms during and 4 months following cryptosporidiosis as compared to 4 months before illness onset

Symptoms	Before illness (baseline)	During illness				After illness			
	*n* (%)	*n* (%)	aOR	*p*	95% CI	*n* (%)	aOR	*p*	95% CI
Dizziness	23 (7.5)	80 (26)	5.13	0.000	3.22–8.18	45 (14.6)	2.25	0.000	1.46–3.47
Headache	44 (14.3)	106 (34.4)	3.49	0.000	2.45–4.98	78 (25.3)	2.15	0.000	1.57–2.93
Fatigue	105 (34.1)	239 (77.6)	7.22	0.000	5.13–10.16	156 (50.7)	2.04	0.000	1.58–2.63
Weight loss	37 (12.0)	196 (63.6)	13.58	0.000	8.85–20.84	61 (19.8)	1.82	0.003	1.23–2.70
Diarrhoea	101 (32.8)	269 (87.3)	15.71	0.000	10.30–23.96	129 (41.9)	*1.50	0.005	1.13–2.01
Loss of appetite	73 (23.7)	248 (80.8)	16.40	0.000	11.08–24.27	91 (29.6)	1.38	0.055	0.99–1.92
Abdominal pain	97 (31.5)	245 (79.6)	9.69	0.000	6.71–13.99	118 (38.3)	*1.38	0.026	1.04–1.83
Joint pain	32 (10.4)	63 (20.5)	2.39	0.000	1.56–3.66	52 (16.9)	1.84	0.001	1.27–2.67
Eye pain	11 (3.6)	30 (9.8)	3.13	0.001	1.59–6.17	19 (6.2)	1.83	0.060	0.97–3.45
Vomiting	25 (8.1)	100 (32.5)	5.72	0.000	3.60–9.09	21 (6.8)	0.83	0.517	0.46–1.47

Number of cryptosporidiosis cases (*n*) and percentage of the total (*n* = 308) number of cryptosporidiosis cases reporting each symptom. Odds ratios (aOR) and corresponding 95% confidence intervals (CI) are adjusted for age, gender, *Cryptosporidium* species, study year and season

*No longer statistically significant when removing children under 5 years of age from the analysis: abdominal pain OR 1.32 (95% CI 0.98–1.79, *p* = 0.071), diarrhoea OR 1.30 (95% CI 0.94–1.80, *p* = 0.108)

Compared to the controls (Table 2), all symptoms but headache, joint pain and eye pain were significantly more likely to be reported among cryptosporidiosis cases 4 months after infection, but this was also true for the period before infection (with the exceptions of dizziness, joint and eye pain). During the acute phase of infection, all symptoms were significantly more likely to be reported among cases than controls (Table 2).

**Table 2:** Occurrence of symptoms 4 months before, during and 4 months after cryptosporidiosis as compared to the control group

Symptoms	Controls (baseline)	Before illness				During illness				After illness			
	*n* (%)	*n* (%)	aOR	*p*	95% CI	*n* (%)	aOR	*p*	95% CI	*n* (%)	aOR	*p*	95% CI
Dizziness	106 (5.9)	23 (7.5)	1.57	0.099	0.92–2.67	80 (26)	8.30	0.000	5.54–12.42	45 (14.6)	3.37	0.000	2.05–5.51
Headache	359 (20.1)	44 (14.3)	0.65	0.028	0.45–0.95	106 (34.4)	2.34	0.000	1.73–3.16	78 (25.3)	1.23	0.236	0.87–1.75
Fatigue	516 (28.8)	105 (34.1)	1.44*	0.011	1.09–1.91	239 (77.6)	11.53	0.000	8.40–15.84	156 (50.7)	2.85	0.000	2.12–3.83
Weight loss	51 (2.9)	37 (12.0)	4.94	0.000	2.99–8.16	196 (63.6)	70.33	0.000	45.42–108.91	61 (19.8)	8.50	0.000	5.11–14.14
Diarrhoea	60 (3.4)	101 (32.8)	32.32	0.000	19.40–53.84	269 (87.3)	564.12	0.000	298.92–1064.62	129 (41.9)	46.07	0.000	26.99–78.62
Loss of appetite	242 (13.5)	73 (23.7)	2.80	0.000	1.66–3.19	248 (80.8)	35.54	0.000	24.73–51.10	91 (29.6)	3.22	0.000	2.28–4.56
Abdominal pain	432 (24.1)	97 (31.5)	1.58	0.002	1.18–2.13	245 (79.6)	15.97	0.000	11.46–22.27	118 (38.3)	2.22	0.000	1.63–3.02
Joint pain	225 (12.6)	32 (10.4)	0.78	0.262	0.50–1.21	63 (20.5)	1.97	0.000	1.37–2.82	52 (16.9)	1.35	0.145	0.90–2.04
Eye pain	110 (6.2)	11 (3.6)	0.78	0.477	0.40–1.54	30 (9.8)	2.40	0.000	1.48–3.89	19 (6.2)	1.32	0.361	0.73–2.41
Vomiting	66 (3.7)	25 (8.1)	2.69	0.000	1.58–4.57	100 (32.5)	15.25	0.000	10.16–22.90	21 (6.8)	1.96*	0.031	1.06–3.61

Number (*n*) of cases or controls and percentage of the total number of cases (*n* = 308) or controls (*n* = 1790) reporting each symptom. Odds ratios (aOR) and corresponding 95% confidence intervals (CI) are adjusted for age, gender, study year and season

*No longer statistically significant when removing children under 5 years of age from the analysis: fatigue before illness OR 1.32 (95% CI 0.96–1.81, *p* = 0.085), vomiting after illness OR 1.89 (95% CI 0.82–4.36, *p* = 0.137)

When data was analysed for the two species separately (Table 3), the most common symptoms 4 months after illness were the same as for both species combined (Table 1**)** or when both species combined were compared to the controls **(**Table 2**)**. Yet amongst cases, none of the symptoms were significantly more likely to occur when infected with *C*. *hominis* vs. *C*. *parvum*, neither before, nor during, nor after illness (Table 4).

**Table 3:** Comparisons of symptom occurrence 4 months before, during and 4 months after cryptosporidiosis and in the control group according to *Cryptosporidium* species

Symptoms	Controls	Before illness				During illness							After illness						
		vs. controls					vs. before illness				vs. controls			vs. before illness				vs. controls	
*C*. *hominis*	*n* (%)	*n* (%)	aOR	*p*	95% CI	*n* (%)	aOR	*p*	95% CI	aOR	*p*	95% CI	*n* (%)	aOR	*p*	95% CI	aOR	*p*	95% CI
Dizziness	106 (6)	7 (8)	2.93	0.022	1.17–7.37	22 (24)	6.40	0.000	2.57–15.99	14.41	0.000	7.01–29.60	13 (14)	2.48	0.073	0.92–6.69	6.04	0.000	2.77–13.19
Headache	359 (20)	13 (14)	0.64	0.184	0.32–1.24	31 (34)	3.63	0.000	1.85–7.13	2.46	0.001	1.42–4.21	22 (24)	2.06	0.027	1.09–3.90	1.35	0.308	0.76–2.41
Joint pain	516 (29)	6 (7)	0.71	0.457	0.28–1.76	13 (14)	2.71	0.033	1.08–6.77	1.85	0.087	0.92–3.74	14 (15)	3.01	0.018	1.21–7.50	2.00	0.049	1.00–3.99
Fatigue	51 (3)	31 (34)	1.63	0.054	0.99–2.69	67 (73)	5.90	0.000	3.15–11.05	9.39	0.000	5.55–15.88	46 (50)	2.07	0.004	1.27–3.38	3.28	0.000	2.04–5.29
Weight loss	60 (3)	9 (10)	*4.95	0.000	2.03–12.10	54 (59)	15.44	0.000	6.12–38.97	73.77	0.000	35.78–152.13	22 (24)	3.03	0.005	1.39–6.62	12.68	0.000	6.13–26.24
Diarrhoea	242 (14)	38 (41)	97.59	0.000	38. 8–245.6	81 (88)	11.81	0.000	5.47–25.47	2828.9	0.000	637.4–12,555.5	46 (50)	1.45	0.158	0.87–2.44	111.83	0.000	47–266.07
Loss of appetite	432 (24)	25 (27)	2.23	0.004	1.29–3.86	76 (83)	14.74	0.000	7.43–29.27	33.26	0.000	17.78–62.23	36 (39)	1.77	0.026	1.07–2.95	4.41	0.000	2.63–7.42
Abdominal pain	225 (13)	32 (35)	1.64	0.053	0.99–2.71	73 (79)	9.10	0.000	4.62–17.93	13.98	0.000	7.92–24.68	42 (46)	1.68	0.076	0.95–2.97	2.75	0.000	1.70–4.46
Eye pain	110 (6)	1 (1)	0.27	0.203	0.04–2.03	7 (8)	9.01	0.036	1.16–69.70	*1.97	0.000	0.81–4.80	4 (4)	4.57	0.223	0.4–52.56	1.10	0.869	0.37–3.29
Vomiting	66 (4)	8 (9)	*2.52	0.023	1.13–5.63	30 (33)	5.44	0.000	2.60–11.40	12.25	0.000	6.42–23.40	7 (8)	0.86	0.765	0.33–2.28	1.60	0.315	0.64–4.04
*C*. *parvum*																			
Dizziness	106 (6)	16 (7)	1.33	0.402	0.69–2.56	58 (27)	5.10	0.000	2.97–8.75	7.67	0.000	4.70–12.52	32 (15)	2.25	0.001	1.39–3.66	3.09	0.000	1.78–5.36
Headache	359 (20)	31 (14)	0.51	0.005	0.32–0.81	75 (35)	3.48	0.000	2.28–5.31	1.68	0.007	1.15–2.43	56 (26)	2.19	0.000	1.54–3.13	1.11	0.613	0.75–1.65
Joint pain	516 (29)	26 (12)	0.75	0.265	0.45–1.25	50 (23)	2.32	0.001	1.43–3.75	1.72	0.013	1.12–2.64	38 (18)	1.60	0.023	1.07–2.39	1.19	0.454	0.75–1.88
Fatigue	51 (3)	74 (34)	1.21	0.296	0.85–1.71	172 (80)	7.95	0.000	5.22–12.10	10.85	0.000	7.34–16.06	110 (51)	2.03	0.000	1.51–2.70	2.60	0.000	1.86–3.65
Weight loss	60 (3)	28 (13)	5.14	0.000	2.78–9.49	142 (66)	13.70	0.000	8.33–22.52	71.99	0.000	42.72–121.34	39 (18)	1.49	0.095	0.93–2.38	7.80	0.000	4.38–13.87
Diarrhoea	242 (14)	63 (29)	25.22	0.000	13.86–45.9	188 (87)	17.08	0.000	10.33–28.24	521.45	0.000	248.9–1092.5	83 (38)	1.53	0.016	1.08–2.16	40.55	0.000	22.47–73.19
Loss of appetite	432 (24)	48 (22)	2.23	0.000	1.48–3.37	172 (80)	17.89	0.000	10.09–28.86	38.26	0.000	24.24–60.39	55 (25)	1.22	0.380	0.78–1.91	2.70	0.000	1.80–4.05
Abdominal pain	225 (13)	65 (30)	1.53	0.024	1.06–2.22	172 (80)	10.43	0.000	6.67–16.30	18.05	0.000	11.83–27.53	76 (35)	1.28	0.145	0.92–1.78	1.96	0.000	1.37–2.81
Eye pain	110 (6)	10 (5)	1.02	0.961	0.48–2.17	23 (11)	2.68	0.011	1.25–5.76	2.38	0.003	1.34–4.25	15 (7)	1.59	0.164	0.83–3.08	1.48	0.243	0.77–2.87
Vomiting	66 (4)	17 (8)	2.66	0.004	1.37–5.16	70 (32)	5.93	0.000	3.31–10.66	16.66	0.000	10.08–27.54	14 (6)	0.81	0.565	0.39–1.67	2.30	0.021	1.14–4.65

Number (*n*) of cases or controls and percentage of the total number of cases (*C*. *hominis* = 92 and *C*. *parvum* = 216) or controls (*n* = 1790) reporting each symptom. Odds ratios (aOR) and corresponding 95% confidence intervals (CI) are adjusted for age, gender, study year and season

*No longer statistically significant when removing children under 5 years of age from the analysis: weight loss before *C*. *hominis* infection vs. controls OR 4.95 (95% CI 0.90–10.18, *p* = 0.073), vomiting before *C*. *hominis* infection vs. controls OR 2.83 (95% CI 0.90–8.89, *p* = 0.074), eye pain during *C*. *hominis* infection vs. controls OR 2.15 (95% CI 0.81–5.69, *p* = 0.125)

**Table 4:** Comparisons of symptom occurrence among cases 4 months before, during and 4 months after infection with *C*. *hominis* vs. *C*. *parvum*

Symptoms	Before illness					During illness					After illness				
	*C*. *hominis*	*C*. *parvum*	aOR	*p*	95% CI	*C*. *hominis*	*C*. *parvum*	aOR	*p*	95% CI	*C*. *hominis*	*C*. *parvum*	aOR	*p*	95% CI
	*n* (%)	*n* (%)				*n* (%)	*n* (%)				*n* (%)	*n* (%)			
Dizziness	7 (8)	16 (7)	0.56	0.297	0.19–1.65	22 (24)	58 (27)	*0.48	0.063	0.22–1.03	13 (14)	32 (15)	0.61	0.277	0.25–1.49
Headache	13 (14)	31 (14)	0.77	0.550	0.33–1.81	31 (34)	75 (35)	0.70	0.292	0.36–1.36	22 (24)	56 (26)	0.84	0.630	0.41–1.72
Joint pain	6 (7)	26 (12)	0.99	0.993	0.34–2.91	13 (14)	50 (23)	0.87	0.734	0.38–1.97	14 (15)	38 (18)	1.05	0.902	0.46–2.39
Fatigue	31 (34)	74 (34)	0.85	0.606	0.45–1.59	67 (73)	172 (80)	0.99	0.987	0.48–2.06	46 (50)	110 (51)	0.92	0.778	0.51–1.65
Weight loss	9 (10)	28 (13)	1.85	0.205	0.71–4.77	54 (59)	142 (66)	1.35	0.349	0.71–2.52	22 (24)	39 (18)	0.95	0.884	0.45–1.97
Diarrhoea	38 (41)	63 (29)	0.80	0.492	0.43–1.50	81 (88)	188 (87)	1.10	0.834	0.44–2.78	46 (50)	83 (38)	0.83	0.538	0.45–1.51
Loss of appetite	25 (27)	48 (22)	1.13	0.728	0.56–2.27	76 (83)	172 (80)	1.12	0.768	0.52–2.44	36 (39)	55 (26)	0.84	0.596	0.43–1.62
Abdominal pain	32 (35)	65 (30)	1.04	0.918	0.53–2.02	73 (79)	172 (80)	0.86	0.700	0.40–1.85	42 (46)	76 (35)	0.62	0.128	0.34–1.14
Eye pain	1 (1)	10 (5)	1.00	0.993	0.11–8.86	7 (8)	23 (11)	1.20	0.737	0.41–3.54	4 (4)	15 (7)	1.09	0.899	0.28–4.25
Vomiting	8 (9)	17 (8)	1.02	0.975	0.34–3.03	30 (33)	70 (32)	1.34	0.375	0.70–2.54	7 (8)	14 (7)	2.21	0.179	0.69–7.00

Number (*n*) of cases and percentage of the total number of cases (*C*. *hominis* = 92 and *C*. *parvum* = 216) reporting each symptom. Odds ratios (aOR) and corresponding 95% confidence intervals (CI) are adjusted for age, gender, study year and season

*Statistically significant when removing children under 5 years of age from the analysis (OR 0.43, 95% CI 0.19–0.95, *p* = 0.038)

When removing children under 5 years of age from the analysis, there were no major differences in the results, with only a few exceptions indicated in the footnotes of Tables 1, 2, 3, and 4.

## Discussion

In line with previous studies [13–16], our data showed that an acute episode of cryptosporidiosis is associated with long-term sequelae. Indeed, 4 months after diagnosis of cryptosporidiosis, several gastrointestinal and non-gastrointestinal symptoms were still occurring at a higher frequency as compared to the period before the onset of acute illness and to the general population (controls). The odds of having non-gastrointestinal symptoms as sequelae were generally higher than those having gastrointestinal symptoms. Yet, when symptom occurrence among cases after illness was compared to the population controls, it appeared that gastrointestinal sequelae were those occurring significantly more often, and that sequelae like joint pain (which occurred significantly more often among cases after illness as compared to before illness) occurred after illness at a rate that was not significantly different from that observed in the general population. At the analysis of the two species separately, results agree with the overall analysis to a major extent, although it is generally more difficult to draw conclusions due to the low numbers of cases. However, the comparison among cases (*C*. *hominis* vs. *C*. *parvum*) clearly showed that there are no significant differences of one *Cryptosporidium* species causing significantly more gastrointestinal (and the other one more non-gastrointestinal) sequelae, as originally hypothesised. Indeed, one of the studies describing sequelae following cryptosporidiosis [16] found that recurrent gastrointestinal symptoms occurred regardless of species, but not the non-gastrointestinal ones (e.g. joint pain, eye pain, headache, dizziness and fatigue). These symptoms were common in cases with *C*. *hominis* infection, but not in those infected with *C*. *parvum*. The other study found no difference in frequency of persisting symptoms between patients infected with *C*. *parvum* or *C*. *homini* [15]. In this regard, our results show a more homogeneous picture with no clear separation of symptoms between the two species. The other two studies investigating sequelae reported information only on either *C*. *hominis* [14] or *C*. *parvum* [13], but not the two together, and both gastrointestinal and non-gastrointestinal symptoms were found to be associated with infection.

The age and gender distribution of the cases was in line with the one expected, as generally younger and middle-aged groups are most affected by *Cryptosporidium* infection. The explanation for the 26–50-year-old age group being particularly affected by diseases like cryptosporidiosis is that this age group often contains parents and child-carers, which are predominantly females, which are thus more likely to be exposed to *Cryptosporidium* from the children themselves [22]. In our study, cryptosporidiosis in older age groups was also prevalent, as reported before [23].

The frequency of some symptoms during and after the illness shows the natural course of the disease, e.g. loss of appetite and vomiting were very likely to occur during the acute phase of the illness, but much less so afterwards. The same is true for non-gastrointestinal symptoms like joint pain, eye pain and headache which were very prevalent during the infection as a direct consequence of the acute infection itself, for example as a consequence of fever and malaise. However, the number of people still experiencing symptoms of, e.g. diarrhoea, fatigue, dizziness and weight loss in the follow-up is striking. The finding that some symptoms occurred more frequently among cases even *before* illness as compared to what is observed in the population controls might be a sign that people acquiring cryptosporidiosis may be a particular group of the population with a generally increased susceptibility to (gastrointestinal) illness per se (e.g. people with underlying chronic conditions), which may also entail an increased likelihood of being diagnosed with cryptosporidiosis due to enhanced medical scrutiny for these patients, as shown for other pathogens [24, 25]. Indeed, high proportions of cases reporting a given symptom after illness had also reported the same symptom before illness (i.e. dizziness 36%, headache 45%, fatigue 54%, weight loss 28%, diarrhoea 50%, loss of appetite 42%, abdominal pain 53%, joint pain 42%, eye pain 32%, vomiting 19%). Results may have also been influenced by substantial recall bias, regarding the symptoms for the period before infection and symptoms occurring after the acute phase were over. Moreover, cases are probably more self-aware due to the initial infection and therefore more prone to remember symptoms after diagnosis. Although there is complete control of between-person confounders, another key limitation of this study design is in the control of within-person confounding, which is still possible for multiple, correlated transient factors that change over time within a subject, such as symptoms related to another acquired illness or injury. Moreover, selection bias may have been introduced by cases being particularly motivated to participate in the follow-up study because of the presence of sequelae. Finally, because the follow-up questionnaire did not emphasise the “chronicity” of sequelae, it was not possible to assess whether symptoms occurred once or several times over the 4-month period after illness.

In conclusion, this study adds to the growing body of evidence for the presence of sequelae following cryptosporidiosis, so far represented by four published studies. While some studies have observed species-specific effects on sequelae, we did not see a clear differentiation between sequelae and the infecting *Cryptosporidium* species. However, subtype information was not available, and there are studies indicating that some subtypes within the same genotype can manifest in different ways and might be more virulent than others [26], which could be interesting to address in future investigations. Although our results do not change the general advice for patient care, awareness of medical personnel should be raised that non-gastrointestinal symptoms can be the consequence of enteric infection. As there is no treatment for cryptosporidiosis, the focus should be on preventive measures.
